# Diversity and Biocontrol Potential of Endophytic Fungi and Bacteria Associated with Healthy Welsh Onion Leaves in Taiwan

**DOI:** 10.3390/microorganisms11071801

**Published:** 2023-07-13

**Authors:** Jian-Yuan Wang, Himanshi Jayasinghe, Yi-Tun Cho, Yi-Chen Tsai, Chao-Ying Chen, Hung Kim Doan, Hiran A. Ariyawansa

**Affiliations:** 1 Department of Plant Pathology and Microbiology, College of Bio-Resources and Agriculture, National Taiwan University, Taipei 106319, Taiwan; geass841215@gmail.com (J.-Y.W.); himanshisachinthana@gmail.com (H.J.); r11633005@ntu.edu.tw (Y.-T.C.); cychen@ntu.edu.tw (C.-Y.C.); 2 Hualien District Agricultural Research and Extension Station, Hualien 973044, Taiwan; yi-chen@hdares.gov.tw; 3 Small Farms & Specialty Crops Advisor, University of California Cooperative Extension, 2980 Washington Street, Riverside, CA 92504, USA; hkdoan@ucanr.edu

**Keywords:** 16S rRNA, antimicrobial peptide genes, curative assay, integrated disease management, ITS, preventive assay

## Abstract

Foliar diseases caused by *Stemphylium* and *Colletotrichum* species are among the major biotic factors limiting Welsh onion production in Taiwan. Owing to concerns about the environment and the development of pathogen resistance to existing fungicides, biological control using endophytes is emerging as an eco-friendly alternative to chemical control. The aim of the present study was to isolate endophytes from healthy Welsh onion leaves and investigate their antagonistic potential against the major phytopathogenic fungi associated with Welsh onion plants in Taiwan. A total of 109 bacterial and 31 fungal strains were isolated from healthy Welsh onion leaves and assigned to 16 bacterial and nine fungal genera using morphological and molecular characterization based on DNA sequence data obtained from nuclear internal transcribed spacer (nrITS) (fungi) and 16S rRNA (bacteria). Evaluation of these endophytic isolates for biocontrol activity against leaf blight pathogens *Colletotrichum spaethianum* strain SX15-2 and *Stemphylium vesicarium* strain SX20-2 by dual culture assay and greenhouse experiments resulted in the identification of two bacterial isolates (GFB08 and LFB28) and two fungal isolates (GFF06 and GFF08) as promising antagonists to leaf blight pathogens. Among the four selected isolates, *Bacillus* strain GFB08 exhibited the highest disease control in the greenhouse study. Therefore, *Bacillus* strain GFB08 was further evaluated to understand the mechanism underlying its biocontrol efficacy. A phylogenetic analysis based on six genes identified *Bacillus* strain GFB08 as *B. velezensis*. The presence of antimicrobial peptide genes (*baer*, *bamC*, *bmyB*, *dfnA*, *fenD*, *ituC*, *mlna*, and *srfAA*) and the secretion of several cell wall degrading enzymes (CWDEs), including cellulase and protease, confirmed the antifungal nature of *B. velezensis* strain GFB08. Leaf blight disease suppression by preventive and curative assays indicated that *B. velezensis* strain GFB08 has preventive efficacy on *C. spaethianum* strain SX15-2 and both preventive and curative efficacy on *S. vesicarium* strain SX20-2. Overall, the current study revealed that healthy Welsh onion leaves harbour diverse bacterial and fungal endophytes, among which the endophytic bacterial strain, *B. velezensis* strain GFB08, could potentially be used as a biocontrol agent to manage the leaf blight diseases of Welsh onion in Taiwan.

## 1. Introduction

Welsh onion (*Allium fistulosum* L.) belongs to the family Alliaceae [1]. This vegetable crop is an important cooking ingredient and traditional medicine in several Eastern countries, including China, Japan, Korea, and Taiwan [2,3,4,5]. One of the major Welsh onion-growing areas in Taiwan is Sanxing Township, in Yilan County, and Welsh onion in Sanxing is famous for the unique flavour of Welsh onion cultivar grown in this area named ‘Si-Ji-Cong’. However, Welsh onion cultivation in Sanxing is severely affected by two major foliar pathogenic fungi: *Stemphylium vesicarium* and *Colletotrichum* spp., causing Stemphylium leaf blight (SLB) and anthracnose, respectively [6]. The current management of foliar diseases of Welsh onion mainly relies on fungicides in Taiwan. Fungicides such as QoIs (Quinone outside inhibitors), SDHIs (Succinate dehydrogenase inhibitors), demethylation inhibitors, and dicarboximide are widely used on *Allium* crops in Sanxing to control foliar diseases, but the amount of fungicide application has continuously increased over time [6]. In a recent study, Wang et al. reported that foliar pathogens, especially *S. vesicarium* strains, showed resistance to fungicides such as strobilurin plus azoxystrobin and kresoxim-methyl that have been used to control SLB in Taiwan [6]. This scenario has been observed in many other countries including the USA and Canada [7]. Moreover, excessive use of certain chemical fungicides has a negative impact on both environmental and human health. Thus, finding alternative approaches is essential to control foliar diseases of Welsh onion.

Applying potential biological-control agents (BCAs) is a powerful tool to control plant pathogens in agricultural systems [8]. The diversity of plant-associated microbes can be explored to identify new effective microorganisms as BCAs [9,10]. Fungi and bacteria naturally occur as endophytes in plants and have been identified as potential BCA candidates to control various plant diseases [11,12]. Endophytes generally protect plants by exhibiting antagonistic behaviour against phytopathogens, which can be direct (hyper-parasitism, production of antibiotics and lytic enzymes) or indirect (inducing systemic resistance, competing for space and nutrients) [13,14,15,16]. In addition, endophytes can improve plant growth through various mechanisms such as fixation of biological nitrogen, solubilization of phosphate and potassium, and production of siderophores [17,18,19]. Moreover, most endophytes can synthesize one or several phytohormones such as auxins, cytokinins, and gibberellins, that can enhance plant growth while moderating the plant hormonal balance [20,21,22]. Several studies have provided the groundwork for controlling foliar diseases of various *Allium* crops such as onion, garlic, and Welsh onion by using BCAs. For example, Zapata-Sarmiento et al. reported that the inoculation of *Trichoderma asperellum* significantly reduced the disease severity of SLB on onions (*A. cepa*) [23]. Roylawar et al. showed that applying *Piriformospora indica* significantly reduced SLB severity in onions by inducing systemic resistance [24]. Similarly, it has been found that the application of potential BCAs can suppress *Colletotrichum* species causing anthracnose in onion. For instance, Galindez et al. demonstrated that three *Trichoderma* species exhibited significant antifungal activity against *C. gloeosrioides* under in vitro conditions [25].

*Allium* species are an abundant source of endophytic microorganisms including bacteria and fungi with many beneficial properties to plants, such as growth promotion and pathogen control [26,27]. For instance, Murtado et al. isolated 40 endophytic bacteria from onions (*A. cepa*) and among them, bacterial strain BBP5.2 exhibited promising inhibition of *Colletotrichum* sp., one of the major foliar pathogens of onions in Brebes, Central Java [28]. In another study, the endophytic bacterium *Serratia plymuthica* isolated from Chinese leek (*A. tuberosum*) significantly reduced the growth of *Botryosphaeria dothidea* causing apple ring rot in post-harvest apples [29]. Furthermore, by both in vitro and in vivo experiments, Ratnawati et al. demonstrated that three endophytic *Trichoderma* strains (T1FLS, T3RZR, and T2RZS) isolated from shallot (*A. cepa* var *agregatum*) in the Palu Valley, showed significant inhibitory activity against *Alternaria porri*, the pathogen of shallot purple blotch disease [30].

Welsh onion also harbours numerous endophytes that can be used to develop various BCAs [31,32,33]. For instance, Sasaki et al. demonstrated that *Streptomyces* sp. TP-A0569 isolated from Welsh onion stem produced fistupyrone, which significantly inhibited infection by *Alternaria brassicicola* in Chinese cabbage [34]. In a recent study, Rashad et al. indicated that endophytic *Bacillus amyloliquefaciens* isolated from garlic plants together with arbuscular mycorrhizal fungi can reduce the severity and incidence of white rot of garlic caused by *Sclerotium cepivorum* by inducing the activity of defence-related enzymes [35]. Nevertheless, studies related to the use of beneficial endophytes of Welsh onions against phytopathogens are still limited in Taiwan. Thus, beneficial endophytic microorganisms with biocontrol potency should be identified so they can be used as an alternative and eco-friendly method to control phytopathogens of Welsh onions.

Eco-friendly management strategies to control major foliar diseases of Welsh onions are lacking in Taiwan. In the present study, we hypothesized that cultivable endophytic microbes associated with leaves of healthy Welsh onion plants may have great potential for biocontrol potency against emerging phytopathogens of Welsh onions. Thus, experiments were designed to (i) isolate and identify fungal and bacterial endophytes inhabiting Welsh onions; (ii) evaluate their potential antagonism against the major leaf blight pathogens of Welsh onions in vitro and in planta; and (iii) determine the potential mechanisms underlying disease suppression.

## 2. Materials and Methods

### 2.1. Fungal Stains and Plant Materials

Pathogenic fungal strains, *Colletotrichum spaethianum* strain SX15-2 and *Stemphylium vesicarium* strain SX20-2, were isolated during our previous studies from infected Welsh onion plants with leaf blight symptoms [6]. For inoculation experiments, the healthy and mature Welsh onion seedlings (70–90 days after planting) were obtained from Welsh onion fields in Wan-Fu Village, Sanxing, Taiwan.

### 2.2. Endophyte Isolation

Based on our previous study and preliminary results, two commercial Welsh onion fields in Sanxing, Taiwan (24°40′50.8″ N 121°40′04.9″ E and 24°41′36.4″ N 121°40′46.2″ E) that were mostly affected by leaf blight fungal pathogens *Stemphylium vesicarium* and *Colletotrcihum spaethianum* were chosen for this study. In total, five samples of healthy Welsh onion plants of Si-Ji-Cong cultivar at fourth-true-leaf stage, not showing any apparent disease symptoms, were collected from each field from June to December 2020. The collected plants were packed immediately into sterilized polyethylene bags and transferred to the laboratory within 24 h, and stored at 4 °C prior to isolation. Before isolation from leaves, surface disinfection of leaves was carried out by following the procedure described by Espinoza et al. [36]. In brief, the leaf samples were washed thoroughly with running tap water, followed by soaking in 75% ethanol for 30 s and rinsing in sterile distilled water for one minute [36]. To confirm that the disinfection process was successful, a 0.1 mL aliquot of the water used for the last washing step was spotted on potato dextrose agar (PDA) (supplemented with 100 mg/L ampicillin) and nutrient agar (NA) plates, and incubated under the same conditions in parallel.

Two isolation techniques were performed to isolate endophytic microbes (i) Direct plate impression of tissues: The disinfected leaf tissues were cut into small pieces (1 cm × 1 cm) and placed on different media (five to six tissue segments on one plate) [37]. (ii) Spread and pour plate technique: The disinfected leaf tissues were macerated using sterile mortar and pestle and re-suspended in 5 mL of sterile distilled water [37]. Serial dilution of the macerated tissue was made up to 10^−3^ dilution by taking 1 mL of well-shaken original suspension and adding into 9 mL of sterile distilled water. Aliquots of 100 μL from each dilution were plated on media. NA and tryptic soy agar (TSA) plates were used for bacterial endophyte isolation and incubated at 28 °C for five days [38]. PDA and water agar (WA) plates were used to obtain fungal endophytes and incubated at 25 °C for seven days [23]. The cultures were monitored every day for the growth of endophytes and each emerging colony was sub-cultured to NA or PDA, and brought into pure culture by single colony isolation.

All strains isolated in this study were initially re-inoculated to Welsh onion plants at the fourth-true-leaf stage to observe whether they caused any visible necrotic lesions on healthy plant leaves. For each isolate, three replicated plants were used. The isolates that caused lesions on leaves were removed and were not used for further studies. For bacterial strains, 20 μL of bacterial suspensions were inoculated on leaves and the inoculated sites of leaves were wrapped with autoclaved cheesecloth. For fungi, 4 mm mycelium plugs were cut from the 7-day-old culture and inoculated on leaves. Later, the sites inoculated with bacterial or fungal strains were wrapped with Parafilm (Bemis^®^, Neenah, WI, USA) to retain moisture. All the plants under treatment were placed in sealed plastic boxes to maintain high humidity, and the cheesecloth and parafilm were removed five days after inoculation. The plants were grown for 12 days, in the growth chamber at 20–25 °C under a 16/8 h light/dark photoperiod to promote disease development.

### 2.3. Identification of Bacterial and Fungal Isolates

For molecular identification of fungal and bacterial isolates, genomic DNA were extracted using an EasyPure genomic DNA kit (Bioman^®^, Bioman Scientific Co., Ltd., New Taipei, Taiwan) following the manufacturer’s protocol. Polymerase chain reactions (PCR) were performed to amplify 16S rRNA of bacteria and ITS of fungi, using universal barcoding primer pairs 27F/1492R and ITS4/ITS5, respectively [39,40]. PCR was conducted in 50 μL microtubes containing 10 ng DNA, 0.8 units Taq polymerase, 1× PCR buffer, 0.2 mL dNTP, 0.3 μL of each primer, and 1.5 mM MgCl_2_. The PCR products were checked for the expected size on 1% agarose gels and sequenced at the Genomics company (New Taipei, Taiwan). All sequences acquired from this study were preliminarily identified to genus level using the BLASTn search engine (http://blast.ncbi.nlm.nih.gov, accessed on 4 April 2021) at the National Center for Biotechnology Information (NCBI).

### 2.4. Antifungal Activity of Endophyte isolates

#### 2.4.1. In Vitro Antagonistic Assay

Antagonistic activity of the isolated endophytes against the major leaf blight pathogens of Welsh onion *C. spaethianum* strain SX15-2 and *S. vesicarium* strain SX20-2 was evaluated by dual culture assay. Agar plugs of each pathogen (4 mm diameter) were placed on one side of PDA. After 24 h of incubation at 25 °C, an endophytic bacterial strain was streaked 4 cm away from the pathogen disk to evaluate the inhibition efficacy of the bacterial strain [41]. For endophytic fungal strains, the mycelial plug of each fungus was placed 4 cm away from the pathogen disk [42]. Control plates were prepared with only the pathogen. The inhibition rate of mycelial growth (IRM) was evaluated using the formula below [6]:

IRM (%) = (Control colony diameter − Treatment colony diameter/Control colony diameter) × 100%

For initial screening, all the non-pathogenic endophytes isolated from Welsh onion leaves were evaluated for their antagonistic ability with duplicates per treatment. Based on the outcome of the dual culture assay, the top four strains with the highest inhibitory activity were selected for the greenhouse pot experiment to evaluate their in planta biocontrol ability.

#### 2.4.2. In Planta Antagonistic Assay

Greenhouse experiments were conducted to test the efficacy of the selected biocontrol candidates on leaf blight pathogens. Bacterial inocula for application on Welsh onions were prepared by culturing bacterial strains in Luria-Bertani (LB) broth (Himedia^®^, Mumbai, India) at 28 °C with 150 rpm shaking overnight and cells were collected by centrifugation (Allegra X-13R Centrifuge, Beckman Coulter, Inc., Brea, CA, USA) at 3250 rpm, 25 °C for 10 min. The supernatant was discarded, and the pellet was re-suspended in sterile distilled water supplemented with 0.1% carboxymethyl cellulose (Showa Chemical Co., Tokyo, Japan) and adjusted to OD_600_ = 1.0 (~1 × 10^8^ cells/mL) using a spectrophotometer [43,44]. For fungal inocula, strains were cultured on PDA for seven days at 25 °C. Cultures were flooded with sterile distilled water combined with 0.05% Tween 20 (Sigma-Aldrich Co., St. Louis, MO, USA), and the resulting suspensions were filtered through sterilized single-layered cheesecloth with a pore size of 100 μm. Concentrations of the conidial suspensions were determined using a haemocytometer and adjusted to 10^6^ spore/mL concentration [45,46]. Preparation of the pathogenic inocula of *C. spaethianum* strain SX15-2 and *S. vesicarium* strain SX20-2 was performed following Wang et al. [6]. The spore suspensions of *S. vesicarium* strain SX20-2 and *C. spaethianum* strain SX15-2 with 0.05% Tween 20 (Sigma-Aldrich Co., St. Louis, MO, USA) were filtered through one-layered cheesecloth and adjusted to 5 × 10^4^ spores/mL and 10^6^ spores/mL, respectively [6,47].

Welsh onion plants at the fourth-true-leaf stage were selected for the experiment and the plant material for the inoculation was prepared following Wang et al. [6]. Welsh onion plants were sprayed until run-off with a suspension of bacterial isolates (OD_600_ = 1.0, 30 mL) and fungal isolates (10^6^ spores/mL, 30 mL) using an airbrush connected to an air compressor (ASAHI Co., Saitama, Japan) at 30 psi. One day after applying biocontrol candidates, plants were inoculated with 30 mL of a spore suspension of the pathogen (5 × 10^4^ spores/mL for, *S. vesicarium* SX20-2 and 10^6^ spores/mL, for *C. spaethianum* strain SX15-2) following the same procedure. The suspension of each biocontrol candidate was re-supplied at three and ten dpi (days after pathogen inoculation). Plants were kept in sealed plastic boxes for five dpi to boost disease development. Plants inoculated with the pathogen and sterile distilled water containing 0.05% Tween-20 were used as positive and negative controls, respectively [48]. Plants were grown at 20–25 °C under natural sunlight in the greenhouse during the entire process. The inoculated leaves were photographed and recorded at 12 dpi. Diseased leaf areas were measured using ImageJ software (http://rsbweb.nih.gov/ij/, accessed on 28 June 2021) and diseased leaf area (DLA) was calculated as follows [6]:

DLA (%) = (Diseased leaf area of the oldest two leaves/The surface area of the oldest two leaves) × 100%

The experiment was repeated in two independent trials with four replicated plants per treatment.

### 2.5. Phylogeny-Based Identification of the Bacterial Biocontrol Candidates

To correctly identify the bacterial endophytes with the highest biocontrol potential, a phylogenetic tree was generated using maximum likelihood (ML). In total, six gene regions including gyrase subunit A (*gyrA*), heat-shock protein groEL (*groEL*), DNA polymerase III subunit alpha (*polC*), phosphoribosylaminoimidazolecarboxamide formyltransferase (*purH*), RNA polymerase subunit B (*rpoB*), and 16S rRNA were amplified to show the phylogenetic relationships of the bacterial endophytes following Rooney et al. and Dunlap [49,50]. NCBI BLASTn was initially used to find the closest matches in GenBank, and the sequences of the closely related matches were downloaded from GenBank following recent publications [51,52] (Table S3). Multiple sequence alignment was performed using MAFFT version 7 (https://mafft.cbrc.jp/alignment/server/, accessed on 12 April 2023). The evolutionary model of each gene locus was evaluated using MEGA v. 7.0.26. A ML analysis with 1000 bootstrap replicates was constructed using raxmlGUI v. 1.5b [53]. The resulting phylogenetic trees were visualized in FigTree v. 1.4.0 (http://tree.bio.ed.ac.uk/software/figtree/, accessed on 12 April 2023).

### 2.6. Biocontrol Potential of Bacillus Velezensis GFB08

Out of the four isolates showing promising results during the antagonistic assays, *Bacillus velezensis* strain GFB08, which had the highest antagonist potential from the greenhouse experiment, was selected to further investigate its mechanisms underlying biocontrol efficacy.

#### 2.6.1. Inhibition of Fungal Mycelial Growth by Extracellular Metabolites

In an attempt to understand the mechanism involved in the in vitro interaction, the secondary metabolites produced by *B. velezensis* strain GFB08 were extracted and evaluated for their antibiosis effect on the radial growth of *C. spaethianum* strain SX15-2 and *S. vesicarium* strain SX20-2 using a cell-free filtrate assay as described by Jeong et al. [54]. In brief, bacterial isolates were grown in a shaker incubator (28 °C) at 180 rpm for 3 days. Subsequently, the supernatant was obtained and centrifuged at 4000 rpm for 10 min at room temperature followed by filtration through a sterile membrane with 0.22 μm pore size to obtain cell-free culture filtrate. The cell-free filtrate was added to a warm PDA medium (60 °C) in a fixed ratio (1:1). The PDA medium mixed with LB only was used as the control. Mycelial plugs of *C. spaethianum* strain SX15-2 and *S. vesicarium* strain SX20-2 were placed in the centre of the agar plate and incubated at 25 °C. After seven days of incubation, the radial mycelial growth of the pathogens was measured, and the morphological change in the mycelium was observed under the microscope (Olympus^®^ BX51, Olympus Co., Tokyo, Japan). IRM was calculated using the same formula as in Section 2.4.1.

#### 2.6.2. Detection of Proteolytic, Cellulolytic, and Chitinolytic Activity

The proteolytic activity was determined using skimmed milk agar (Himedia^®^, Mumbai, India). The bacterial suspension (10 μL) was placed on the medium and incubated at 25 °C for two days. Protease production was identified by the formation of a clear zone around colonies [55].

The cellulase enzyme activity test was performed using a medium containing 1% peptone, 1% yeast extract, 1% carboxymethyl cellulose, 0.5% sodium chloride, 0.1% monopotassium phosphate, and 1.6% agar (pH 7) [56]. The bacterial suspension (10 μL) was placed on the centre of the medium and incubated at 28℃. After two days of incubation, the plates were flooded with Congo red solution (5 mg/mL, Sigma-Aldrich Co., St. Louis, MO, USA). The clear zone around the colony indicated a positive result for cellulase production.

Chitin detection media was prepared by following the protocol described by Agrawal and Kotasthane [57]. Colloidal chitin and indicator dye bromocresol purple were combined to prepare the media for testing chitin production. Plates containing *B. velezensis* GFB08 were incubated at 28 ± 2 °C for five days. The appearance of colour changes from yellow to purple nearby the colony showed a positive result for chitinase production.

Five replicates were used for each experiment and each experiment was repeated in two independent trials.

#### 2.6.3. Analysis of Antibiotic Biosynthesis Genes

*B. velezensis* strain GFB08 was characterized for the presence of antibiotic biosynthesis genes (*bac*, *baer*, *bamC*, *bmyB*, *dfnA*, *fenB*, *fenD*, *ituC*, *ituD*, *mlna, mycC*, and *srfAA*) using specific primers as listed in Table S2 [58,59,60,61,62].

### 2.7. Preventive and Curative Action

To evaluate the preventive and curative action of *B. velezensis* strain GFB08, the strain was applied (30 mL, OD_600_ = 1.0) on Welsh onion plants one day prior (preventive) and one day after (curative) inoculation with each pathogen (10^6^ spores/mL for *C. spaethianum* strain SX15-2 and 5 × 10^4^ spores/mL for *S. vesicarium* strain SX20-2). Strain GFB08 was re-applied three and ten dpi following the methods illustrated above. A fungicide mixture of pyraclostrobin and boscalid (Wonderful^®^, Sigma-Aldrich Co., St. Louis, MO, USA) was used as the positive control. Plants inoculated with sterile distilled water containing 0.05% Tween-20 and 0.1% carboxymethyl cellulose (Showa Chemical Co., Tokyo, Japan) were used as the negative controls. Diseased leaf areas were measured by ImageJ and DLA was calculated by the methods described above.

The experiment was repeated in two independent trials with four replicated plants per treatment.

### 2.8. Statistical Analysis

Statistical analysis was performed with the R statistical software version 4.2.2 [63]. Student’s *t*-test (*α* = 0.05) was used to compare the means of pathogen mycelial growth inhibition by extracellular metabolites of *B. velezensis* GFB08. Data for dual culture assays, in planta assays and extracellular enzyme assays were analysed using one-way analysis of variance (ANOVA), followed by Tukey’s HSD (honestly significant difference) test (*p* ≤ 0.05) for mean separation.

## 3. Results

### 3.1. Field Survey and Endophyte Isolation

**Figure 1:**
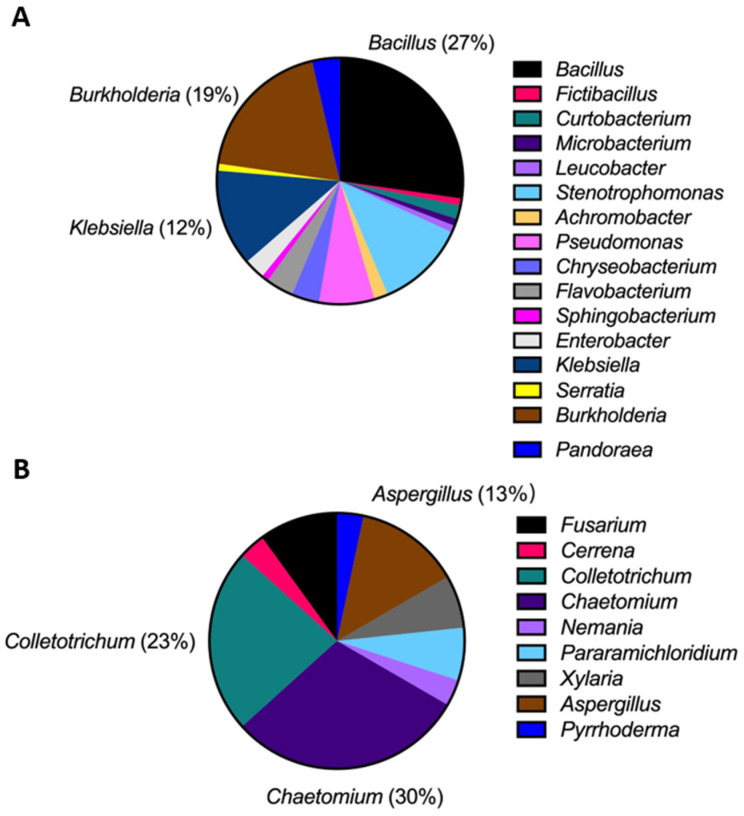
Composition of endophytes isolated from healthy Welsh onion leaves at the genus level. (**A**) Proportion of bacterial endophytes. (**B**) Proportion of fungal endophytes.

A total of 109 bacterial and 31 fungal strains were isolated from the leaves of healthy Welsh onion plants. Isolated strains were classified into taxonomic groups based on DNA sequence data of ITS (fungi) and 16 rRNA (bacteria). Based on the BLASTn results, endophytic strains were grouped into sixteen bacterial and nine fungal genera. Among the identified bacterial genera, *Bacillus*, *Burkholderia*, and *Klebsiella* were the most dominant, representing 27%, 19%, and 12% of the total, respectively. Among the fungal isolates, *Chaetomium* (30%), *Colletotrichum* (23%), and *Aspergillus* (13%) were identified as the most dominant genera (Figure 1).

### 3.2. Dual Culture and Pot Assays for the Selection of Promising BCAs

#### 3.2.1. Dual Culture Assay

To identify the most promising BCAs for further study and to understand their potential biocontrol mechanisms, several screening experiments were conducted and the strains without significant biocontrol potential were eliminated. Strains were selected as follows. Initially, all the strains isolated from Welsh onion leaves were inoculated to healthy Welsh onion plants at the four-leaf stage to check whether they caused any necrotic lesions. Based on the initial screening, nine strains were identified as pathogenic isolates as they caused visible necrotic lesions on healthy leaves; these were excluded from further analysis.

**Figure 2:**
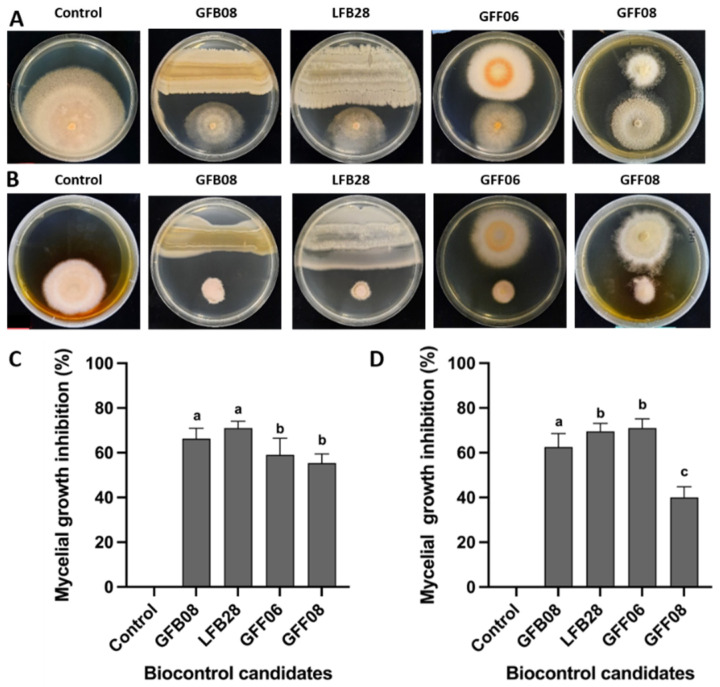
Inhibition of pathogen mycelial growth by biocontrol candidates (dual culture assay). (**A**,**C**) *C. spaethianum* SX15-2. (**B**,**D**) *S. vesicarium* SX20-2. Control, cultures with pathogen only. Columns represent means of four technical repeats and two biological repeats and the vertical bars indicate standard error. Columns with different letters are significantly different according to Tukey’s HSD (*p* ≤ 0.05).

Out of 131 non-pathogenic endophytes, four strains (GFB08, LFB28, GFF06 and GFF08, Table S1) that exhibited significant inhibitory activity against leaf blight pathogens in dual culture assay were selected for further investigation. Out of the four strains, *Bacillus* strains GFB08 and LFB28 showed the highest inhibitory activity against *C. spaethianum* strain SX15-2 by reducing the mycelial growth rate up to 66% and 71%, respectively. Compared to *Bacillus* strains, two fungal strains (*Fusarium* GFF06 and *Chaetomium* GFF08) exhibited moderate activity against *C. spaethianum* strain SX15-2, respectively, reducing the mycelial growth rate by 59% and 56% (Figure 2A,C). With *S. vesicarium* strain SX20-2, *Bacillus* strains GFB08 and LFB28 exhibited inhibitory activities of 63% and 70%, respectively, while fungal strains *Fusarium* GFF06 and *Chaetomium* GFF08 exhibited inhibitory activities of 71% and 40%, respectively (Figure 2B,D).

#### 3.2.2. Disease Suppression under Greenhouse Conditions

**Figure 3:**
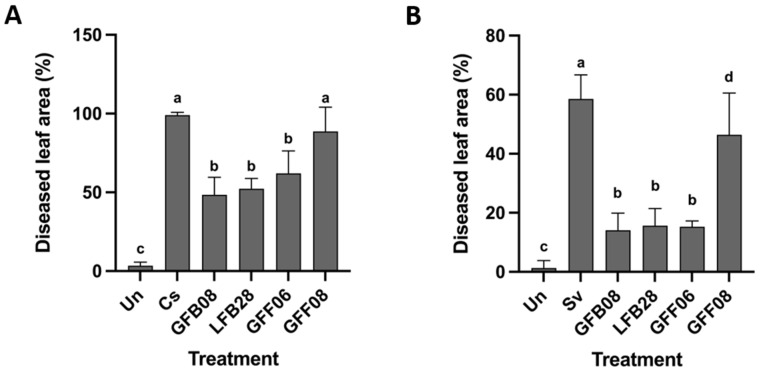
Disease suppression assay of potential BCAs on foliar pathogens under greenhouse conditions. (**A**) *C. spaethianum* strain SX15-2. (**B**) *S. vesicarium* strain SX20-2. Un, un-inoculated plants; Cs, plants inoculated with *C. spaethianum* strain SX15-2 only; Sv, plants inoculated with *S. vesicarium* strain SX20-2 only. Columns represent means of four technical repeats and two biological repeats and the vertical bars indicate standard error. Columns with different letters are significantly different according to Tukey’s HSD (*p* ≤ 0.05).

The results of the greenhouse study suggested that the application of *Bacillus* strains (LFB28 and GFB08) and *Fusarium* GFF06 reduced the DLA caused by *C. spaethianum* strain SX15-2 up to 52%, 48%, and 62%, respectively (Figure 3A). The DLA caused by *S. vesicarium* strain SX20-2 decreased up to 15%, 14%, and 15% after the application of *Bacillus* (LFB28 and GFB08) and *Fusarium* GFF06, respectively (Figure 3B). The application of *Chaetomium* GFF08 did not show a significant reduction in infection rate compared to the positive control when the plant was inoculated with *C. spaethianum* strain SX15-2 and *S. vesicarium* strain SX20-2. Based on the results of this in planta assay, *Bacillus* GFB08 strain was considered the most promising BCA and used for further studies.

### 3.3. Identification of Bacillus Biocontrol Candidates

**Figure 4:**
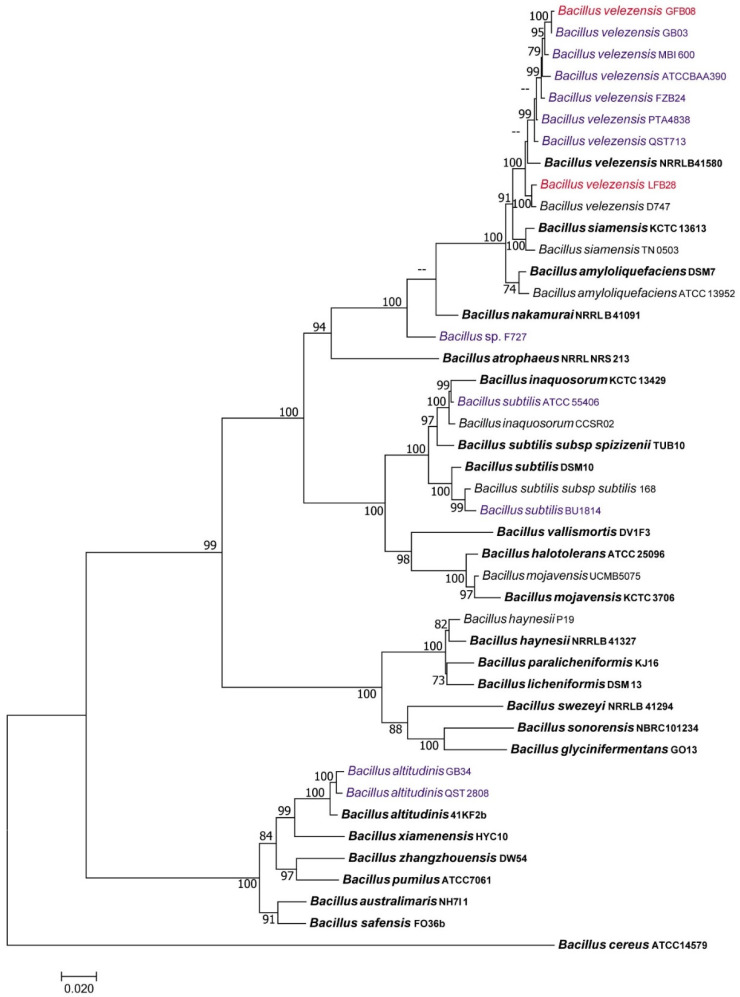
Maximum-likelihood (GTR+G+I model) phylogenetic tree of *Bacillus subtilis* group based on six genes (16S, *groEL*, *gyrA*, *polC*, *purH*, and *rpoB*). BS greater than 70% are marked at the nodes. Isolates obtained in the present study are in red, the ex-type sequences are indicated in bold, and registered commercial *Bacillus* strains are in purple. *Bacillus cereus* ATCC 14579 was used as the outgroup. The scale bar shows the number of estimated mutations per site.

*Bacillus* isolates (LFB28 and GFB08) were further analysed to determine their correct taxonomic identity. Several datasets were organized to infer phylogenies of bacterial strains based on ML analysis. The strains selected for the phylogenetic analysis were based on Dunlap [49]. The dataset consisted of 5560 characters including genes encoding *gyrA*, *groEL*, *polC*, *purH*, *rpoB*, and 16S rRNA. A best scoring RAxML tree is shown in Figure 4, with the likelihood value of −37,688.462084. The ML tree obtained from this study showed overall topologies of species level relationships in agreement with previous work based on ML [50]. The two most promising *Bacillus* strains used in this study formed a well-supported clade within the clade containing the ex-type strain *B. velezensis* NRRL B-41580. Therefore, the *Bacillus* strains (LFB28 and GFB08) were identified as *B. velezensis*.

### 3.4. Biocontrol Potential of B. velezensis GFB08

#### 3.4.1. Effect of Extracellular Metabolites of *B. velezensis* GFB08 on Mycelium Growth

**Figure 5:**
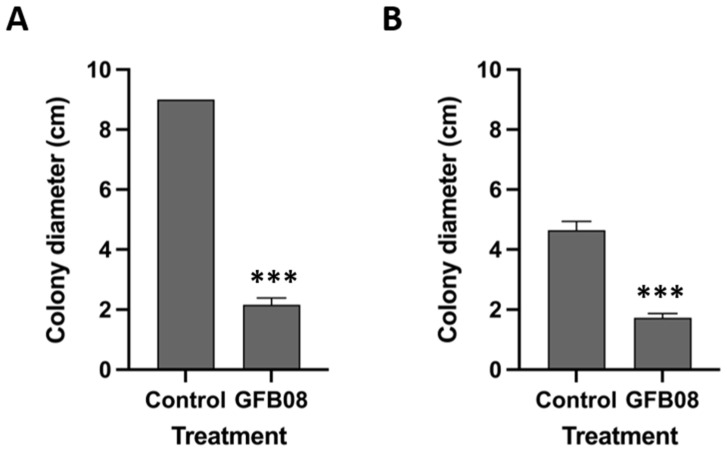
The inhibitory effect of cell culture filtrates of *B. velezensis* GFB08 on colony diameter of foliar pathogens. (**A**) *C. spaethianum* SX15-2 (Cs). (**B**) *S. vesicarium* SX20-2 (Sv). Data are presented as means and standard error of four technical replicates and two biological repeats. Means labelled with asterisks are significantly different (*p* < 0.05) compared with the control according to student’s *t* test. (***, *p* < 0.001).

As mentioned previously, *B. velezensis* strain GFB08 showed significant inhibitory effects on mycelium growth of both *C. spaethianum* strain SX15-2 and *S. vesicarium* strain SX20-2 (Figure 2). To determine whether the suppression of the pathogens was dependent on toxic metabolites, culture filtrate of *B. velezensis* strain GFB08 was assessed for its effects on mycelium growth of both pathogens. Cell free culture filtrate from *B. velezensis* GFB08 significantly inhibited the mycelium growth of both *C. spaethianum* strain SX15-2 and *S. vesicarium* strain SX20-2 (Figure 5 and Figure S1). Moreover, hyphae and conidia of *C. spaethianum* strain SX15-2 became swollen and distorted when grown on medium mixed with filtrate. Unlike *C. spaethianum* strain SX15-2, hyphae of *S. vesicarium* strain SX20-2 did not show any significant difference in morphology compared to the control. However, cell free filtrate of *B. velezensis* strain GFB08 significantly reduced mycelium growth and spore germination of *S. vesicarium* strain SX20-2 (Figure S1). The results of the cell free filtrate assay suggest that the antagonistic mechanisms of *B. velezensis* strain GFB08 against *C. spaethianum* strain SX15-2 and *S. vesicarium* strain SX20-2 may be related to extracellular metabolites produced by *B. velezensis* GFB08.

#### 3.4.2. Extracellular Enzyme Activity of *B. velezensis* GFB08

Hydrolytic enzyme tests of protease, cellulase, and chitinase were performed to check the extracellular enzymatic activity of *B. velezensis* strain GFB08. *B. velezensis* strain GFB08 produced protease and cellulase, but not chitinase (Figure S2).

#### 3.4.3. Detection of Antibiotic Coding Genes in *B. velezensis* GFB08

**Figure 6:**
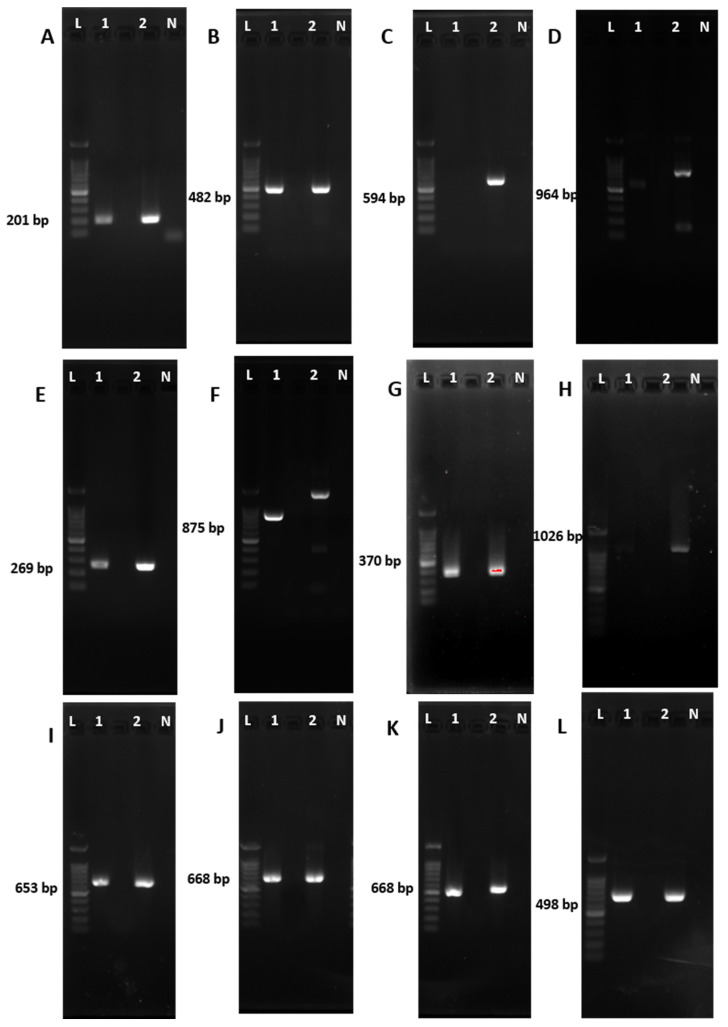
Detection of antibiotic biosynthesis genes in *B. velezensis* strain GFB08. (**A**) *srfAA*; (**B**) *ituC*; (**C**) *ituD*; (**D**) *fenB*; (**E**) *fenD*; (**F**) *bamC*; (**G**) *bmyB*; (**H**) *mycC*; (**I**) *dfnA*; (**J**) *mlna*; (**K**) *baer*. (**L**) *bac*. Lane L, Omic Bio 100 bp DNA ladder; Lane 1, *B. velezensis* GFB08; Lane 2, *B. velezensis* LFB28; Lane N, negative control.

Specific primer pairs encoding genes for the biosynthesis of dipeptides, lipopeptides, and polyketides (Table S2) were used to determine the presence of antibiotic biosynthesis genes of *B. velezensis* strain GFB08. The amplification results suggested that *B. velezensis* strain GFB08 is able to synthesize antibiotics such as bacillaene, bacillomycin, bacilysin, difficidin, fengycin, iturin, macrolactin, and surfactin (Figure 6). The presence of genes encoding the above antibiotics might indicate their involvement in the mechanism of suppressing the growth of both *C. spaethianum* strain SX15-2 and *S. vesicarium* strain SX20-2.

### 3.5. Preventive and Curative Action of B. velezensis GFB08

**Figure 7:**
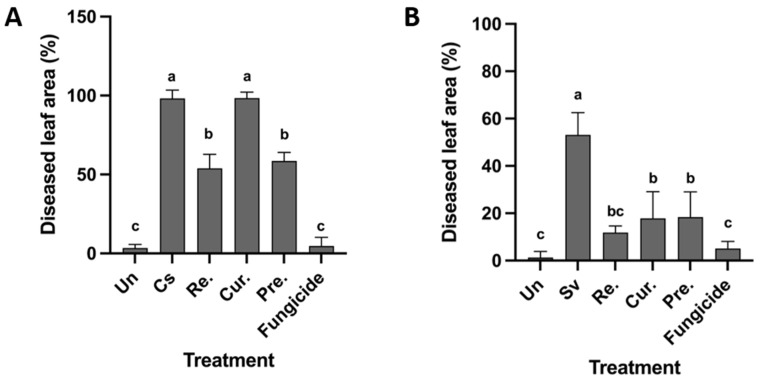
Disease suppression by *Bacillus velezensis* strain GFB08 under greenhouse conditions. (**A**) *C spaethianum* strain SX15-2. (**B**) *S. vesicarium* strain SX20-2. Un, uninoculated plants; Cs, plants inoculated with *C. spaethianum* strain SX15-2 only; Sv, plants inoculated with *S. vesicarium* strain SX20-2 only; Re, cell suspension of *B. velezensis* GFB08 was applied on leaves of Welsh onion one day prior to inoculation with the pathogen and re-applied two times at three and ten dpi; Cur, cell suspension of *B. velezensis* strain GFB08 was applied on leaves of Welsh onion one day after inoculation with pathogen; Pre, cell suspension of *B. velezensis* strain GFB08 was applied on leaves of Welsh onion one day prior to inoculation with the pathogen; Fungicide, mixture of pyraclostrobin plus boscalid was applied one day after pathogen inoculation. Columns represent means of four technical repeats and two biological repeats and the vertical bars indicate standard error. Columns with different letters are significantly different according to Tukey’s HSD (*p* ≤ 0.05).

The results of preventive and curative activity of *B. velezensis* strain GFB08 against leaf blight pathogens showed that the application of strain GFB08 one day prior to pathogen inoculation reduced disease severity of leaf blight caused by *C. spaethianum* strain SX15-2 by up to 58%. However, the application of strain GFB08 one day after inoculation with the same pathogen did not significantly reduce disease severity (Figure 7A). Both preventive and curative treatments of strain GFB08 on leaves reduced disease severity caused by *S. vesicarium* strain SX20-2 up to 18% and 17%, respectively (Figure 7B). A common fungicide used in Welsh onion fields for foliar pathogens (pyraclostrobin + boscalid) was also tested to compare the efficacies of biocontrol candidates and chemical fungicide. Applying fungicide, respectively, reduced 96% and 95% of disease severity caused by *C. spaethianum* strain SX15-2 and *S. vesicarium* strain SX20-2. The results of this experiment suggest that *B. velezensis* strain GFB08 exhibits preventive effects on *C. spaethianum* while exhibiting both preventive and curative effects on *S. vesicarium* strain SX20-2.

## 4. Discussion

Control of plant diseases using beneficial microbes is an environmentally friendly and important component of integrated pest management (IPM). Endophytic microbes residing in host plants are valuable natural resources that can be exploited as BCAs due to their beneficial effects on development, growth, and fitness of the host plant [64,65]. Although Welsh onion is an economically important vegetable crop in many countries, research exploring its endophytic communities is lacking. In the present study, antagonistic potential of bacterial and fungal endophytes isolated from healthy Welsh onion leaves were evaluated for their antagonistic potential against major foliar pathogens of Welsh onion.

In the present study, the majority of the fungal strains isolated from healthy Welsh onion leaves belonged to the genus *Chaetomium* (Figure 1B). Several previous studies indicate that *Chaetomium* species can occur as endophytes of *Allium* crops and show inhibitory activity against plant pathogens. For instance, *C. globosum* isolated from *A. sativum* showed significant inhibitory activity against *Fusarium oxysporum*, which causes basal rot in onion [66]. The second most abundant fungal genus was *Colletotrichum*, accounting for 23% of the fungal strains isolated from healthy Welsh onion plants (Figure 1B). *Colletotrichum* contains numerous phytopathogenic species and has been reported from various *Allium* crops causing anthracnose on leaves and smudge on bulbs worldwide [67,68]. The lifestyles of *Colletotrichum* species can be categorized as necrotrophic, hemibiotrophic, latent or quiescent, and endophytic [69]. Prusky et al. defined quiescence (latency) as a continued period in the fungal life cycle in which the pathogen remains dormant within the plant host before it switches to an active phase [70]. During latency, activity of the pathogen is almost suspended. The quiescent stage in *C. truncatum* after inoculation to *Capsicum annuum* fruit was reported by Ranathunge et al. to lack apparent symptoms until six dpi [71]. Thus, the results of the present study suggest that *Colletotrichum* strains isolated from Welsh onion plants without symptoms might be related to the quiescent behaviour of the *Colletotrichum* species associated with host plants.

*Bacillus* species account for the majority (27%) of the bacterial strains isolated in the present study (Figure 1A). *Bacillus* species have been reported as endophytes of *Allium* crops [72,73]. According to Wang et al., *B. siamensis* isolated from *A. sativum* bulbs significantly inhibited the white rot disease caused by *Sclerotium cepivorum* while promoting plant growth [16]. In the present study, isolates exhibiting the highest biocontrol potential against foliar pathogens also belonged to the genus *Bacillus*. *Burkholderia*, the second most predominant bacterial genus, includes approximately 19% of the total endophyte isolates. *Burkholderia* species have been isolated as endophytes from various *Allium* crops [74]. Pellegrini et al. indicated that onion seeds inoculated with a consortium of *B. ambifaria* showed increased plant height and crop yields [75].

Recent studies have found that strains expressing the best activities in vitro are not always the strains showing the best results in planta and vice versa [76]. For example, reports on *B. cereus* isolate BT8 showed a lack of antagonism to *Phytophthora capsici* by in vitro studies, but the same organism suppressed lesion development caused by *P. capsici* on cocoa (*Theobroma cacao*) leaves under field applications [77]. Therefore, in the present study, we used all four isolates with promising results in vitro for the in planta study to select the strain with best biocontrol performance for further studies. The result of the greenhouse assay showed that applying *Bacillus* strains GFB08, GFB28, and *Fusarium* strain GFF06 significantly reduced the disease severity caused by both *C. spaethianum* strain SX15-2 and *S. vesicarium* strain SX20-2 (Figure 3); compared to the other tested isolates, *B. velezensis* strain GFB08 showed the highest control efficacy against both *C. spaethianum* strain SX15-2 and *S. vesicarium* strain SX20-2, even though it did not have the highest inhibition of those pathogens in the dual culture assay. This phenomenon showed that in vitro and in planta results do not always correlate and reflect disease suppression within the same levels. Nonetheless, in vitro studies and their results are particularly useful for identifying likely candidates for biocontrol and for making educated guesses concerning the mechanisms by which they reduce pathogen damage. Finally, *Bacillus* strain GFB08, which showed the highest pathogen control from the greenhouse assay, was selected for further studies including the mechanisms underlying its bio-controlling efficacy.

In the present study, *Bacillus* GFB08 strain was identified as *B. velezensis* in a multi-gene phylogeny based on 16S, *groEL*, *gyrA*, *polC*, *purH*, and *rpoB* gene regions [50]. Dunlap recommended these six gene regions to determine the species limits of the *B. subtilis* species complex, as analysis of the 16S rRNA gene alone is insufficient due to its highly conserved nature. The *B. subtilis* species complex includes *B. amyloliquefaciens*, *B. atrophaeus*, *B. axarquiensis*, *B. malacitensis*, *B. mojavensis*, *B. sonorensis*, *B. vallismortis*, *B. tequilensis*, and *B. velezensis* [78,79,80,81,82,83,84,85]. Most of these species (endophytic or non-endophytic) are well-known plant pathogen antagonists. For instance, a recent study found that *B. amyloliquefaciens* YN201732, a beneficial endophyte isolated from tobacco seeds controlled the pathogenic fungus *Erysiphe cichoracearum* causing powdery mildew in tobacco by inducing defence-related gene expressions [86]. Another study found that an endophytic *B. atrophaeus* strain, DM6120, isolated from *Fragaria* × *ananassa* roots produced volatile inhibitory compounds and lytic enzymes to control the strawberry anthracnose pathogen *Colletotrichum nymphaeae* [87]. Moreover, *B. velezensis* endophyte C2, isolated from the crown tissue of a tomato, significantly reduced Verticillium wilt incidence in tomatoes by secreting antibiotics and lytic enzymes [88].

One of the best known and most important mechanisms used by BCAs to limit pathogen invasion in host plant tissues is antibiosis through the production of anti-pathogen metabolites [89,90]. Strains identified as *B. velezensis* have been shown to exhibit remarkable biocontrol activity against phytopathogens due to the production of the lipopeptide group of antibiotics such as bacillomycin, fengycin, iturin, and surfactin [91]. For example, *B. velezensis* isolated from soil was reported to produce surfactin and bacillomycin D against *Colletotrichum gloeosporioides*, which caused anthracnose on mangoes (*Mangifera indica*) [92,93]. Kim et al. (2021) reported similar findings and identified *B. velezensis* AK-0, a BCA against bitter rot caused by *C. gloeosporrioides* in apples, encoding antimicrobial genes of bacillaene, bacillomycin, bacilysin, difficidin, iturin, macrolactin, and surfactin [93]. In the same study, Kim et al. further reported that *B. velezensis* AK-0 expressed higher levels of *ituD* and *bacD* during interaction with pathogenic *C. gloeosporrioides* and reduced the disease severity [94]. Based on PCR in the present study, *B. velezensis* GFB08 encodes genes of bacillaene, bacillomycin, bacilysin, difficidin, fengycin, iturin, macrolactin, and surfactin, consistent with recent findings related to *B. velezensis* (Figure 6). Presence of these genes indicated that the antagonistic effect might be due to the secretion of certain antifungal metabolites by *B. velezensis* strain GFB08 against *C. spaethianum* strain SX15-2 and *S. vesicarium* strain SX15-2. However, the presence of these genes does not guarantee that they are expressed during the interactions with pathogens. Therefore, further studies based on qRT-PCR should be conducted to check this hypothesis of whether these genes are expressed during the interaction between pathogen and BCA.

In addition to lipopeptides, *B. velezensis* is well-known for its production of CWDEs. For example, Shin et al. demonstrated that *B. velezensis* HYEB5-6 inhibited the disease development of *C. gloeosporioides* on *Euonymus japonicus* by producing cellulase and protease [95]. The in vitro enzyme tests in the present study indicated that *B. velezensis* strain GFB08 could produce several CWDEs including cellulase and protease (Figure S2). This property can play an important role in the natural environment, allowing the BCA to degrade the cell wall material of pathogenic fungi. Therefore, secretion of these enzymes indicated that the antagonistic effect might also be related to the production of certain CWDEs by *B. velezensis* strain GFB08 against *C. spaethianum* strain SX15-2 and *S. vesicarium* strain SX15-2.

This is the first study investigating the diversity of bacterial and fungal endophytes harboured in the leaves of healthy Welsh onions in Taiwan. Moreover, this is the first report showing the biocontrol efficacy of the naturally occurring endophyte *B. velezensis* strain GFB08 in controlling leaf blight fungal pathogens associated with Welsh onions. The findings of this study are significant because the diversity of Welsh onion endophytes has not been fully explored and the possibility of employing Welsh onion endophytes as BCAs against Welsh onion foliar diseases has not been studied before.

## 5. Conclusions

In the present study, 109 bacterial and 31 fungal endophytic strains were isolated from healthy Welsh onion leaves in fields with leaf blight diseases. The results indicated that among the endophyte isolates, two bacterial isolates (GFB08 and LFB28) and two fungal isolates (GFF06 and GFF08) could significantly inhibit leaf blight pathogens under both in vitro and in planta conditions. Among the four antagonists tested in the greenhouse assay, *B. velezensis* strain GFB08 had the highest control of the disease by reducing the lesion area caused by *Colletotrichum spaethianum* strain SX15-2 and *Stemphylium vesicarium* strain SX20-2 up to 48% and 14%, respectively. Various mechanisms might be involved in biocontrol activity against leaf blight pathogens, such as production of antimicrobial compounds and CWDEs. Taken together, the results of this study reveal that *B. velezensis* strain GFB08 can be developed as a BCA to control and manage Welsh onion leaf blight diseases. However, further studies should be carried out under field conditions to evaluate its biocontrol efficacy, effect on plant growth, influence on indigenous microbial communities as well as the effect of agronomic practices (chemical fertilizers, pesticides, fungicides, etc.) on *B. velezensis* strain GFB08. 

## Data Availability

The data presented in this study are available on request from the corresponding author.

## Supplementary Materials

The following supporting information can be downloaded at: https://www.mdpi.com/article/10.3390/microorganisms11071801/s1, Figure S1: The inhibitory effect of cell free filtrates of *B. velezensis* GFB08 on mycelium growth and spore germination of *C. spaethianum* SX15-2 (Cs) and *S. vesicarium* SX20-2 (Sv); Figure S2: Production of extracellular enzymes by *B. velezensis* GFB08; Table S1: Strain ID and genus names of the biocontrol candidates exhibited promising results in antagonistic assays; Table S2: Primers used to detect the presence of genes synthesizing different antibiotics (bacilisyne, bacillaene, bacillomycin, difficidin, fengycin, iturin, macrolactin and surfactin) in *B. velezensis* GFB08; Table S3: *Bacillus* strains and GenBank accession numbers of DNA sequences used in the phylogenetic study.
