# Supplementary material for: Diversity and Biocontrol Potential of Endophytic Fungi and Bacteria Associated with Healthy Welsh Onion Leaves in Taiwan
Source: Microorganisms. 2023 Jul 13;11(7):1801. doi: 10.3390/microorganisms11071801 (PMC10386586; doi:10.3390/microorganisms11071801)
Supplement: Supplementary file 1 [file microorganisms-11-01801-s001.zip › microorganisms-2444856-supplementary.pdf]

Supplementary data

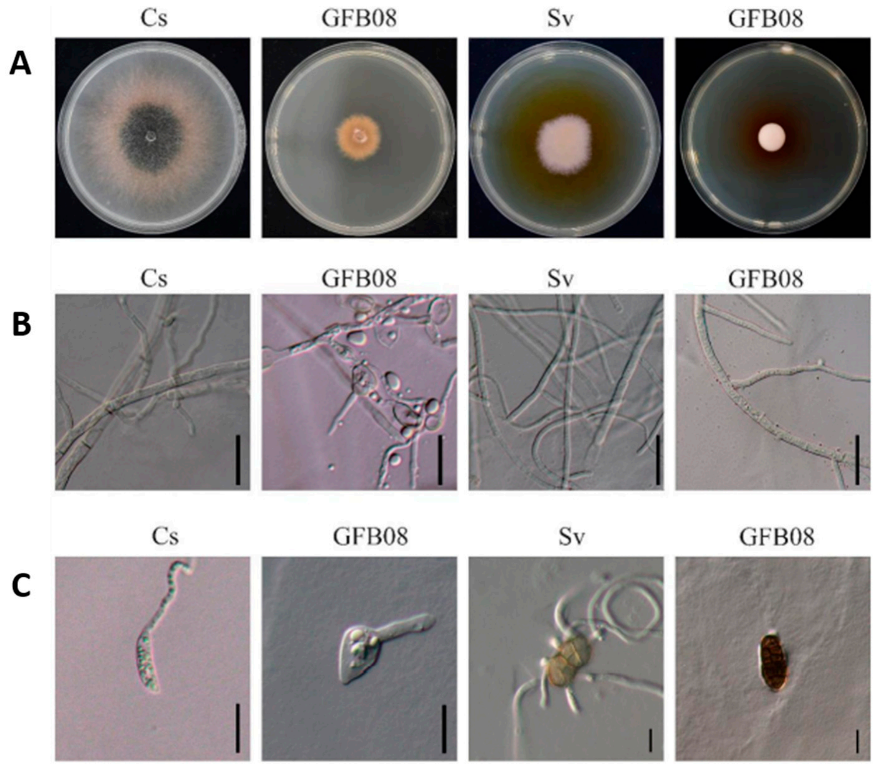

Figure S1. The inhibitory effect of cell free filtrates of *B. velezensis* GFB08 on mycelium growth and spore germination of *C. spaethianum* SX15-2 (Cs) and *S. vesicarium* SX20-2 (Sv). **(A)** Mycelium growth of pathogens after seven days of incubation at 25°C. **(B)** Microscopic examination of hyphae after incubation at 25°C for seven days. **(C)** Microscopic examination of spore after incubation at 25°C for 24 hours. Cs, *C. spaethianum* SX15-2. Sv, *S. vesicarium* SX20-2. Scale bars: B = 20 μm, C = 10 μm.

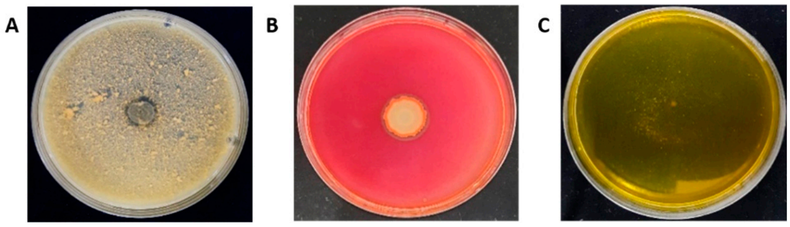

Figure S2. Production of extracellular enzymes by *B. velezensis* GFB08. **(A)** protease; **(B)** cellulase; **(C)** chitinase.

Table S1. Strain ID and genus names of the biocontrol candidates exhibited promising results in antagonistic assays.

**Strain ID**	**Genus**
GFB08	*Bacillus*
LFB28	*Bacillus*
GFF06	*Fusarium*
GFF08	*Chaetomium*

Table S2. Primers used to detect the presence of genes synthesizing different antibiotics (bacilisyne, bacillaene, bacillomycin, difficidin, fengycin, iturin, macrolactin and surfactin) in *B. velezensis* GFB08.

**Antibiotics**	**Genes**	**Primers**	**Sequences** (5'-3')	**Product size** (bp)	**Annealing temperature**	**References**
Bacilisyne	*bac*	BAC-F<br>BAC-R	CAGCTCATGGGGCTTTT<br>CTCGGTCCTGAAGGGACAG	498	60 ºC	Mora et al., 2011
Bacillaene	*baeR*	BAER-F<br>BAER-R	ATGTCAGCTCAGTTTCCGCA<br>GATCGCCGTCTTCAATTGCC	668	56 ºC	Compaoré et al., 2013
Bacillomycin	*bamC*	Bacc1-F<br>Bacc1-R	GAAGGACACGGAGAGAGTC<br>CGCTGATGACTGTTCATGCT	875	60ºC	Ramarathnam et al., 2007
Bacillomycin	*bmyB*	BMYB-F<br>BMYB-R	GAATCCCGTTGTTCTCCAAA<br>GCGGGTATTGAATGCTTGTT	370	55 ºC	Chung et al., 2008

Difficidin	*dfnA*	DFna-F<br>DFna-R	GGATTCAGGAGGGCATAC<br>G<br>ATTGATTAAACGCGCCGAG<br>C	653	56 ºC	Compaoré et al.,<br>2013
Fengycin	*fenB*	FENB2F<br>FENB2R	CAAGATATGCTGGACGCTGA<br>ACACGACATTGCGATTGGTA	964	62 ºC	Ramarathnamet al.,<br>2007
Fengycin	*fenD*	FEND-F<br>FEND-R	GGCCCGTTCTCTAAATCCAT<br>GTCATGCTGACGAGAGCAAA	269	58ºC	Mora et al.,<br>2011
Iturin	*ituC*	ITUC-F1<br>ITUC-R1	TTGAAYGTCAGYGCSCCTTT<br>TGCGMAAATAATGGSGTCGT	482	55 ºC	Chung et al.,<br>2008
Iturin	*ituD*	ITUD-F1<br>ITUD-R1	CCCCCTCGGTCAAGTGAATA<br>TTGGTTAAGCCCTGATGCTC	594	53 ºC	Chung et al.,<br>2008
Macrolactin	*mlna*	Mlna-F<br>Mlna-R	CCGTGACGGAC<br>TGGATGAG<br>CATCGCACCTGCCAAATAC<br>G	668	56 ºC	Compaoré et al.,<br>2013
Micosubtilin	*mycC*	MYCC-F<br>MYCC-R	AATCAATTGGCACGAACCT<br>T<br>ATCGCCCGTTTTGTACATT<br>C	1026	55 ºC	Chung et al.,<br>2008
Surfactin	*sfp*	Sfp-F<br>Sfp-R	ATGAAGATTTACGGAATTTA	675	58ºC	Hsieh et al.,<br>2004

			TTATAAAAGCTCTTCGTACG			
Surfactin	*srfAA*	SRFA-F<br>SRFA-R	TCGGGACAGGAAGACATCAT<br>CCACTCAAACGGATAATCCTGA	201	55ºC	Mora et al., 2011

Table S3. *Bacillus* strains and GenBank accession numbers of DNA sequences used in the phylogenetic study.

Species	Strain number	GenBank accession numbers					
		**16S**	***gyrA***	***groEL***	***polC***	***purH***	***rpoB***
B. amyloliquefaciens	DSM7*	FN597644.1	FN597644	FN597644.1	FN597644.1	FN597644.1	FN597644.1
B. amyloliquefaciens	ATCC 13952	CP009748.1	CP009748.1	CP009748.1	CP009748.1	NZ CP009748.1	NZ CP009748.1
B. altitudinis	QST 2808	MK613092.1	MK608735	MK608723	MK608747	MK608759	MK608771
B. altitudinis	GB34	MK613093.1	MK608736	MK608724	MK608748	MK608760	MK608772
B. altitudinis	41KF2b*	NR042337.1	ASJC01	ASJC01000009.1	ASJC01000001	ASJC01000009.1	NZASJC01000012.1
B. altitudinis	41KF2b*	NR042337.1	ASJC01	ASJC01000009 .1	ASJC01000001	ASJC01000009.1	NZASJC01000012.1
B. atrophaeus	NRRL-NRS-213*	NR116190.1	LSBB01	EU138585.1	LSBB01000020.1	NZLSBB01000023.1	NZLSBB01000009.1
B.	NH7I-1*	MW228046.1	NZLGYN01000023.1	LGYN01000007.1	ASJD01000003.1	NZLGYN01000031.1	NZLGYN01000003.1

australimaris							
B. cereus	ATCC 14579*	NR114582.1	NR114582.1	CP034551.1	CP034551.1	NC004722.1	NC004722.1
B. glycinifermentans	GO-13*	KT005408.1	LECW02	LECW02000020.1	KR092206.1	NZLECW02000020.1	KR092212.1
B. halotolerans	ATCC 25096*	MN840041.1	NZLPVF01000008.1	PVF01000011.1	LPVF01000010.1	NZLPVF01000011.1	NZLPVF01000014.1
B. haynesii	NRRL B-41327*	NZMRBL01000076.1	MRBL01	-	MRBL01000005.1	NZMRBL01000006.1	NZMRBL01000043.1
B. haynesii	P19	NZCP059494.1	CP059494.1	CP059494.1	CP059494.1	NZCP059494.1	NZCP059494.1
B. inaquosorum	KCTC 13429*	NZAMXN01000021.1	AMXN01	-	CP029465.1	NZAMXN01000005.1	NZAMXN01000011.1
B. inaquosorum	CCSR02	NZCP080644.	NZCP080644.1	CP080644.1	CP080644.1	NZCP080644.1	NZCP080644.1
B. licheniformis	DSM 13*	MN117660.1	CP000002	AE017333.1	AE017333.1	AE017333.1	AE017333.1
B. mojavensis	KCTC 3706*	AB021191.1	NZAYTL01000028	-	EU138667.1	NZAYTL01000011.1	EF015389.1
B. mojavensis	UCMB5075	CP051464.1	CP051464.1	CP051464.1	CP051464.1	CP051464.1	CP051464.1
B. nakamura	NRRL-B	NR151897.1	LSAZ01	LSAZ01000021.1	LSAZ01000041.1	NZLSAZ01000023.1	NZLSAZ01000009.1

i	41091*						
B. paraliche niformis	KJ-16*	NR137421.1	LBMN02	LBMN02000015.1	LBMN02000035.1	NZLBMN02000023.1	NZLBMN02000017.1
B. pumilus	ATCC 7061 *	NR043242.1	ABRX01000004	EU138586.1	ABRX01000001.1	ABRX01000003.1	EU138862.1
B. safensis	FO-36b*	MW 228825.1	CP010405	CP010405.1	CP010405.1	ASJD01000006.1	KC895451.1
B. siamensis	KCTC 13613*	MW578390.1	AJVF01	-	-	NZAJVF0100002 3.1	KC608574.1
B. siamensis	TN 0503	KY777345.1	KY777250.1	KY777326.1	KY777307.1	KY777288.1	KY777269.1
B. sonorensis	NBRC 101234 *	NR113993.1	AYTN01	EU138542.1	EU138680.1	NZBCVZ0100000 7.1	NZBCVZ0100002 0.1
Bacillus sp.	F727	MK613086.1	MK608729	MK608717	MK608741	MK608753	MK608765
B. spizizenii	TUB10 *	CP002905	CP002905	CP002905.1	CP002905.1	CP002905.1	CP002905.1
B. subtilis	ATCC 55406	MK613088.1	MK608731	MK608719	MK608743	MK608755	MK608767
B. subtilis	BU1814	MK613094.1	MK608737	MK608725	MK608749	MK608761	MK608773
B. subtilis	168	NR102783.2	AL009126	CP010052.1	CP010052.1	NC000964.3	NC000964.3
B. swezeyi	NRRL B-41294*	NR 157608	MRBK01	QSNF01000005.1	MRBK01000006.1	NZMRBK01000040.1	NZMRBK01000022.1
B. vallismortis	NRRL B-14890*	NZJH600273.1	EU138601.1	EU138532.1	EU138670.1	NZJH600241.1	NZJH600199.1

B. velezensis	QST 713	MK613083.1	MK608726	MK608714	MK608738	MK608750	MK608762
B. velezensis	ATCC BAA 390	MK613084.1	MK608727	MK608715	MK608739	MK608751	MK608763
B. velezensis	FZB24	MK613085.1	MK608728	MK608716	MK608740	MK608752	MK608764
B. velezensis	D747	MK613087.1	MK608730	MK608718	MK608742	MK608754	MK608766
B. velezensis	PTA 4838	MK613089.1	MK608732	MK608720	MK608744	MK608756	MK608768
B. velezensis	GB03	MK613090.1	MK608733	MK608721	MK608745	MK608757	MK608769
B. velezensis	MBI 600	MK613091.1	MK608734	MK608722	MK608746	MK608758	MK608770
B. velezensis	NRRL B-41580*	KY694464.1	LLZC01	LLZC0100002 2.1	EU138691.1	EU138760.1	EU138829.1
B. velezensis	**GFB08**	**-**	**-**	**-**	**-**	**-**	**-**
B. velezensis	**LFB28**	**-**	**-**	**-**	**-**	**-**	**-**
B. xiamensis	HYC-10*	NR148244.1	AMSH01	AMSH01000005.1	AMSH01000001	AMSH01000005	NZAMSH01000009.1
B. zhangzhouensis	DW5-4*	NR148786.1	JOTP01	JOTP01000006.1	JOTP01000002.1	NZJOTP01000006.1	NZJOTP01000017.1

Notes: ATCC American Type Culture Collection, Manassas, VA, USA; NRRL Agricultural Research Service Culture Collection, National Center for Agricultural Utilization Research, US Department of Agriculture, Peoria, IL, USA. KCTC Korean Collection for Type Cultures, Genetic Resources Center, Korea Research Institute of Bioscience and Biotechnology, Taejon, Republic of Korea; DSM Deutsche Sammlung von

Mikroorganismen und Zellkulturen GmbH, Braunschweig, Germany. Newly isolated strains in this study are indicated in bold. Type sequences are annotated with asterisks after the strain number
